# Estimating Spatial and Temporal Trends in Environmental Indices Based on Satellite Data: A Two-Step Approach

**DOI:** 10.3390/s19020361

**Published:** 2019-01-17

**Authors:** Brigitte Colin, Kerrie Mengersen

**Affiliations:** School of Mathematical Sciences, Queensland University of Technology, Brisbane, QLD 4000, Australia; k.mengersen@qut.edu.au

**Keywords:** boosted regression tree, spatio-temporal analysis, fractional cover data, prediction of location-based vegetation trends

## Abstract

This paper presents a method for employing satellite data to evaluate spatial and temporal patterns in environmental indices of interest. In the first step, linear regression coefficients are extracted for each area in the image. These coefficients are then employed as a response variable in a boosted regression tree with geographic coordinates as explanatory variables. Here, a two-step approach is described in the context of a substantive case study comprising 30 years of satellite derived fractional green vegetation cover for a large region in Queensland, Australia. In addition to analysis of the entire image and timeframe, separate analyses are undertaken over decades and over sub-regions of the study region. The results demonstrate both the utility of the approach and insights into spatio-temporal trends in green vegetation for this site. These findings support the feasibility of using the proposed two-step approach and geographic coordinates in the analysis of satellite derived indices over space and time.

## 1. Introduction

Remotely sensed data are available from a wide range of sources, ranging from satellites to drones, and have been used for a very wide range of environmental applications and analysis of spatial and temporal trends. For example, Landsat data are freely available [1]; the imagery covers a wide geographical area, and it avoids expensive, extensive and often impractical in situ measurement. There is a strong advantage in using remotely sensed Landsat imagery for land use and land cover (LULC) analyses in detecting and estimating the magnitude of spatio-temporal trends in measures of the quantity of green vegetation [2,3]. Monitoring long-term trends of green vegetation in a semi-arid region gives valuable insight into dependencies and changing quantities influenced by climate variability. For monitoring and analysis of green vegetation, the infrared (IR) and near infrared (NIR) spectral channels are best suited since they discriminate between green and active vegetation versus woody vegetation or organic litter [4]. Fractional cover (FCover) data is a derived product out of Landsat imagery and shows the fractions of existing land cover in one pixel as percentages that are contained within the pixel. A satellite pixel combines the reflected radiation from different objects on the earth surface, and this spectral mixing effect results in a so called mixed pixel, or Mixel [5]. In a spectral unmixing approach, the Landsat pixel is divided into assigned biophysical variables [6,7,8,9]. For example, in our study described below, we used only the band that shows the fractions for green vegetation out of a three-layer composite containing two additional layers for bare soil and for non-green vegetation. The derivation of FCover is described in [6,10,11].

Environmental Modelling is important when we want to understand and monitor the local variability and spatial trends over time of green vegetation. Instead of applying our method to the full resolution, we used aggregated FCover pixels showing a much coarser resolution to examine green vegetation trends. The aggregation scheme, the best resolution for LULC studies and its suitability are described in our paper [12]. The goal is to detect spatial and temporal trends based on 30 years of data. More specifically, we want to understand how the linear trend in FCover changes over the spatial region, and how well this can be described by geographic coordinates. For this, we specify qualitative factor levels showing six categories of green vegetation trends that are listed and further described in Table 2. Then, we model these trends using latitude and longitude coordinates that serve as a North–South gradient (latitude) and East–West gradient (longitude).

The use of latitude and longitude as surrogate covariates is not uncommon. For example, in a study in [13], the authors used latitude and longitude coordinates as surrogate variables for North–South and East–West gradients to account for the variation in deciduous forested ecoregions. The response was an aggregated Normalized Difference Vegetation Index (NDVI) variable used as an on-site quantification of vegetation in North America. Similarly, in a study of the geographic distribution of plant functional types [14], the authors examined the relationship of precipitation and temperature on C3 (cool-season grasses) and C4 (more drought resistant warm-season grasses) grass types and shrubs using latitude and longitude coordinates. Along a given longitude, C3 grasses increased with latitude and as one moved westward, C4 grasses were replaced by shrubs. They concluded that latitude and longitude can be used as surrogate variables for the main climatic dimensions of the area. The latitude and longitude explained a substantial portion of the variability of the distribution of the relative abundance of shrubs, C3 grasses, and C4 grasses.

In general, there are many methods using machine learning approaches for predicting temporal and spatio-temporal trends that are not limited to green vegetation. Examples include long-term seasonal changes of the Danube River eco-chemical status [15], epidemiology studies and analysis of disease processes in public health [16], spatial and temporal trends of birds over France [17], long-term trends in dryland vegetation variability in Ethiopia [18], and identification of environmental controls in fire-prone biome and spatial patterns at several spatial scales in the Canadian boreal forest [19]. In a previous paper, we evaluated the performance of a popular machine learning technique, namely Boosted Regression Tree (BRT), and concluded that it can perform well in high-dimensional and complex problems, deal with missing data by default without the need for interpolation/infilling, describe complex nonlinearity and interactions between variables, deal with spatial and non-spatial data and different data granularities, and reduce data complexity without negatively affecting prediction performance [20]. However, the focus of that paper was on spatial estimation of environmental indices at a single point in time. In this paper, we focus on estimation over both space and time. We do this by proposing a two-step approach comprising the extraction of slope coefficients out of the model summary and the predictions of the extracted slope coefficients using BRT. The combination of a linear regression and a nonlinear BRT model defines our two-step approach.

The detection of trends in change of green vegetation over time is essential for the assessment of the impacts of climate variability on the LULCC (Land Use Land Cover Change) of a region. The study described in this paper aims to determine the annual trends of slope coefficients over a semi-arid region. Long-term (1987–2017) gridded aggregated FCover fractions of green vegetation data are used to spatially divide the FCover scene. Historical trends are examined using a linear model to regress the aggregated green fraction over time for each grid cell. The extracted grid-specific slope coefficients are then used as a response variable, with the corresponding latitude and longitude as covariates, in the hierarchical supervised machine learning BRT model. The BRT results thus provide an evaluation of the spatial nature of the overall temporal trend in green vegetation over time.

The paper is structured as follows. Section 1 provides background information, places our study in context to other studies and demonstrates why there is a gap we need to fill. Section 2 introduces the study area and presents the context of the other linear model approach to extracting the slope coefficient. In Section 3, we introduce the BRT modelling approach and describe the hyperparameter tuning steps and the model goodness of fit. Section 4 presents the results of the two stages of the analysis. The implications of the data and the output of the prediction of the BRT, as well as strengths and limitation measures of BRT are discussed in Section 5.

## 2. Data Description

### 2.1. Case Study

The FCover scene of our study area is located the Northern Territory, Australia, in the Landsat footprint of path 102 row 72 according to the Worldwide Reference System-2 (WRS-2). The scene covers an area of 185 × 185 km and the elevation is ranging from 50 m to 213 m. The location of our study area is classified as “Dry” with variations of “desert, hot arid” and “dry Summer, hot arid” (BWh and Bsh) based on the Köppen–Geiger scheme and presents heterogeneous and complex topography of native grass types. Arid and semi-arid areas cover a large part of the earth’s surface and are located around the tropics at 23${}^{\circ }$ north and south of the equator. According to the commonly used Köppen–Geiger climate classification, these areas are defined by limited precipitation and high potential evaporation rates. Furthermore, our study area is used for commercial grazing purposes and is highly dependent on grazing practices that ensure future sustainable land use [21].

### 2.2. Fractional Cover Data

There is a strong advantage in using Landsat satellite data for monitoring vegetation trend in land use and land cover (LULC) studies [2,3]. The imagery covers a wide geographical area; it avoids expensive, extensive and often impractical in situ measurement and it is freely available [1]. The spatial resolution of a Landsat pixel combines the reflected or emitted radiation from different objects on the Earth’s surface and, as described in the Introduction above, this spectral mixing effect results in a so-called mixed pixel or Mixel where individual spectra of objects cannot be separated [5]. Fractional cover (FCover) data is a derived product based on Landsat 5 Thematic Mapper (TM) imagery. An extensive ground cover sampling study [10] was used to inform a spectral unmixing algorithm [6,10,11].

### 2.3. Data Pre-Processing

**Figure 1:**
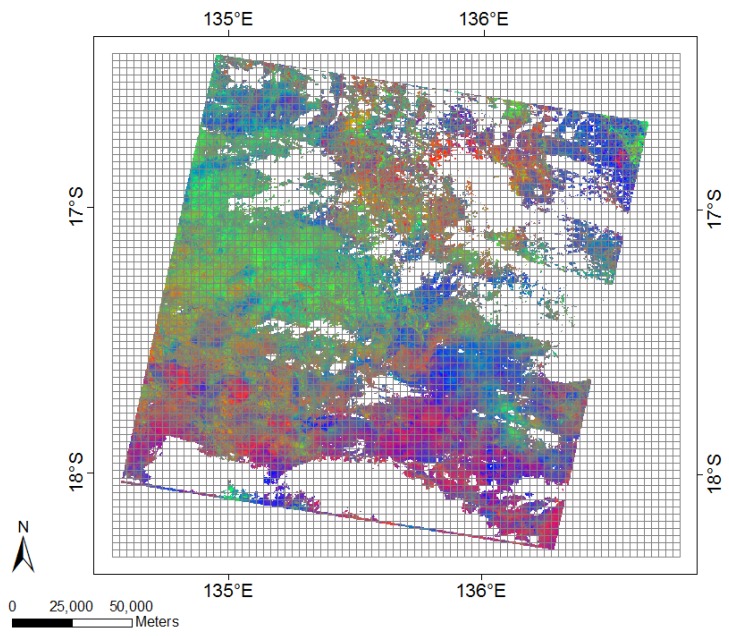
The Fractional Cover (FCover) data are overlaid with an evenly spaced grid where each grid cell contains 100 × 100 pixels and covers an area of 3000 × 3000 m. Each of the total 5530 grid cells will be used to delineate the slope coefficients showing green vegetation trend on unique locations of the spatial grid. The FCover scene shows the relationship of the three ground cover classes of green vegetation (green), non-photosynthetic vegetation (blue) and bare soil (red) referenced on the Worldwide Reference System-2 [12].

In Colin et al. [12], we investigated spatial aggregation schemes that are best suited for this study site with regard to up-scaling FCover data and maintaining sufficient local characteristics for accurate prediction of green vegetation. Instead of dealing with the original amount of 54 million pixels, we thus reduced the data volume to 5530 individual spatial grid cells containing 100 × 100 pixels in each as demonstrated in Figure 1.

**Figure 2:**
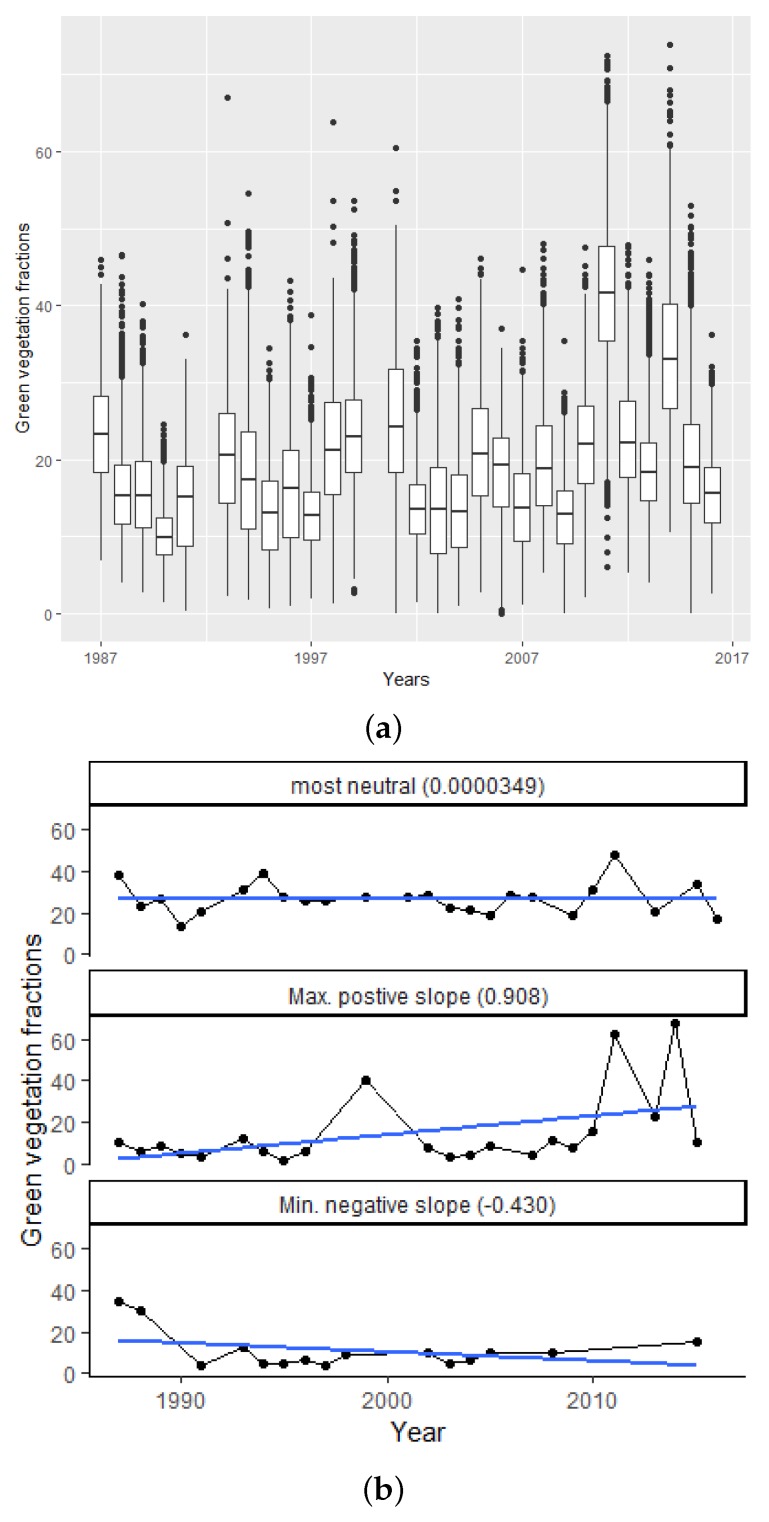
Development of green vegetation fractions and their trends over time shown as boxplots in Figure 2a or as the three most distinctive trends in Figure 2b. (**a**) boxplots showing a strong variation of green vegetation for each year over the 30 year timeframe, 1987–2016. For consistency over time, and because the FCover in the study area is dominated by wet and dry seasons, only December scenes have been used for this case study; (**b**) trend of green vegetation in a 30 years time frame overlaid with a blue linear regression line showing the direction of trends. (top) most neutral, (middle) maximum positive slope and (bottom) minimum negative slope. Only sites with at least 15 observations over the time period were considered for this plot.

Missing data are common in remotely sensed imagery. As part of enhancing data quality, obscuring elements such as clouds and cloud shadows are filtered out, resulting in data gaps. The amount of missing data can be substantial and often imagery can not be used at all due to too many data gaps as demonstrated in Figure 2a in the year 1992 and 2000. Furthermore, Figure 1 shows a FCover scene with an overlaid evenly spaced grid where we can see that we have empty grid cells and data gaps where no information of green vegetation fractions is present.

For studies on marine and vegetation monitoring using remotely sensed imagery from earth observation satellites a geographic scale of 1 km and finer is mostly used, for example MODIS (Moderate-resolution Imaging Spectroradiometer), Landsat and ENVISAT (Environmental Satellite) MERIS (Medium Resolution Imaging Spectrometer). For climate related studies, a coarser spatial resolution is preferred. The Meteosat Second Generation (MSG) deliver data recorded in 12 channels with a spatial resolution of 3 km. MSG data are primarily designed for meteorological observations of the atmosphere, but there are land use applications based on MSG data as well [22,23]. In a particularly interesting investigation [24], the authors used a spatial scale of 3 km to present the first results of NDVI for the entire African continent.

### 2.4. Data Exploration

Summaries of the data extracted from the FCover scenes covering a 30-year time frame for this case study are presented in Table 1 and Figure 2. Table 1 presents overall summary statistics for the observed grid-level values of green vegetation fraction. Figure 2 summarises the spatio-temporal nature of the data, through boxplots of the annual distribution of the grid-level values as well as three trends over time.

## 3. Methods

**Figure 3:**
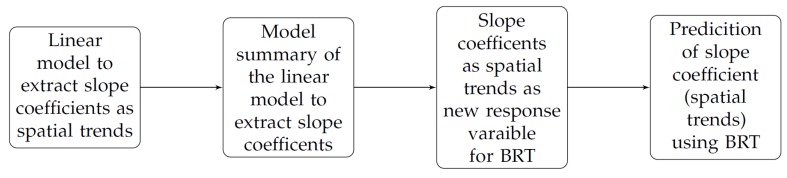
Two-step modelling approach to predict extracted slope coefficients (as spatial trends) using a BRT model.

Our goal for this study is to evaluate the spatial, temporal and spatio-temporal nature of vegetation trends in FCover data. For this, we followed a two-step approach comprising a linear model for the extraction of the slope coefficients (serving as trends in vegetation) followed by the prediction of those extracted slope coefficients using geographic coordinates through a Boosted Regression Tree (BRT) model (see Figure 3).

### 3.1. Linear Model

#### 3.1.1. Extraction of Slope Coefficients

In the first step, for each grid cell, we extracted linear slope estimates from least squares linear regression models for which we used a continuous response variable showing the aggregated fraction of green vegetation and a discrete predictor variable indicating the years from 1987 to 2017. (This was performed using the lm package in R [25], with the formula: green vegetation fractions ∼ years). A positive (negative) slope coefficient indicates an increasing (decreasing) trend and increasing (decreasing) quantity in green vegetation fractions over time. The R software [26] and its basic linear model function was used to fit the model. Each individual grid cell is indexed as *i* and our data set comprise 5530 individual grid cells per year and FCover scene as demonstrated in Figure 1. We further assume that green vegetation, denoted as $Y_{i}$, is linearly related to the covariates year, denoted as *X*, and that the residuals $\epsilon_{i}$ are distributed $N(0,\sigma^{2})$. A linear regression model with one predictor variable can be expressed with the following equation: $Y_{i}=\beta_{0}+\beta *X_{i}+\epsilon_{i}$. The parameters in the model are $\beta_{0}$ as the *Y*-intercept and β as the regression coefficient (the slope coefficient representing the linear trend over time), which we extract from the model summary.

**Figure 4:**
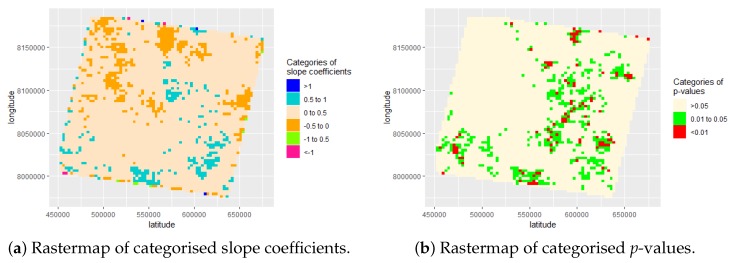
Location of *p*-values and of slope coefficients’ categories.

Table 2 shows the different slope coefficients that were calculated. It can be seen that there are three dominating slope coefficient classes ranging from a negative trend of −0.5 up to a positive trend of 1. Furthermore, we can see that the most extreme values are exclusively found on the outer rim of the rastermap shown in Figure 4a and their overall representation/contribution is marginal (0.02% and 0.03%) as demonstrated in Table 2. We suspect this is based on the natural shift of the Landsat footprint recording which therefore results in extreme data gaps that adversely influence the linear regression analyses and hence the accuracy of the estimates of the corresponding slope coefficients. By visualising all six categories of our extracted slope coefficients, we can see that the three strongest categories ranging from negative −0.5 up to a positive trend of 1, together comprise 99.93% of the slope coefficients as demonstrated in Figure 4a.

The extracted and categorised slope coefficients were plotted using their geographic centroid coordinates from the overlaid spatial grid. This resulted in Figure 4a showing six categories and their location-based slope coefficients based on a 30-year time frame. Furthermore, we can see that the most extreme values are exclusively found on the outer rim of the plot. We suspect this is based on a natural shift of the Landsat footprint and therefore extreme data gaps and in general a lower availability of FCover fractions to sufficiently estimate reliable slope coefficients. Statistically significant trends were assumed to exist if the *p*-values of the slope coefficient were different from zero at a level of 5% (*p* < 0.05) or smaller. The levels of statistical significance for each grid cells are shown in Figure 4b where the *p*-values >0.05 are most common.

**Figure 5:**
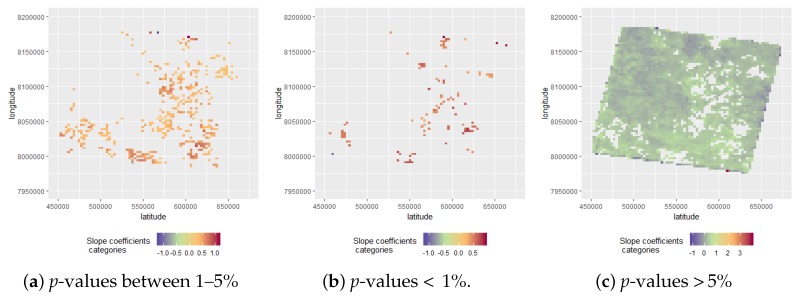
Comparison of the three *p*-value categories with their associated slope coefficients.

Figure 5 shows the extracted three categories of *p*-values, their slope coefficients and associated statistically significant localised effect. We can clearly see that the *p*-values > 5% are most common. In the second step of our two-step approach, we used the estimated slope coefficients for each grid cell as our new response variable in a BRT model to predict green vegetation trends using geographic coordinates. The model is described in detail below.

### 3.2. Boosted Regression Tree

Our research aim was to investigate in spatial, temporal and spatio-temporal trends in green vegetation. The methodological aim was the extension of the created BRT model to predict quantitative long-term trends of green vegetation cover in a semi-arid region that is vulnerable and sensitive to climate variability. For this, we used 30 years of data and a two-step approach comprising the extraction of the slope coefficients (our trends) using a linear model, and used those slope coefficients as our new response variable for the BRT modelling.

**Figure 6:**
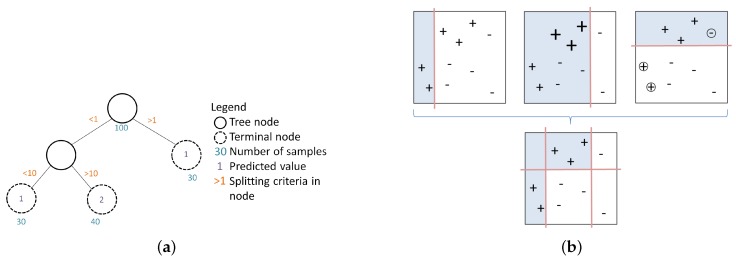
Combination of two algorithms, namely Boosting and linear regression tree within a BRT. (**a**) Regression Trees: hierarchical regression and the binary splitting process showing observations in the nodes, predicted values in the terminal nodes and splitting criteria along the tree branches [12]; (**b**) boosting: BRT as an ensemble approach combines several binary splits to create complex prediction rules that offer more flexibility in dividing the feature space than a single regression tree. Boosting additively fits binary trees and gradually prioritise poorly modelled data to produce a set of binary splits that maximally reduce the BRT loss function, adapted from [12,28].

A BRT is a flexible supervised machine learning method that consists of two algorithms: first, a regression tree approach and, second, boosting. In a regression tree, the feature space is divided in binary trees as shown in Figure 6a, whereas Boosting combines several single binary trees to create a more flexible partition of the feature space Figure 6b. Boosting proceeds by fitting another regression tree to the residuals, until a stopping criteria is reached, or when an acceptable goodness of fit for predictive accuracy is achieved. Details of BRTs are provided in [27].

There is a number of statistical machine learning methods for fitting complex regressions of the type considered here. For example, generalised additive models (GAM) [29] provide a flexible extension of well known generalised linear models and have been widely used in ecological applications, for example to predict tree species in Utah [30]. This author also compared GAM with stochastic gradient boosting (SGB) and found that both had merits with respect to predictive fit. Using another ecological case study, Leathwick et al. [31] compared GAM and BRT and found that BRT showed substantially superior predictive performance. Furthermore, in our paper, we compared three different regression methods (Random Forests and Least Absolute Shrinkage and Selection Operator, LASSO) with each other and evaluated their predictive performance on heterogeneous spatial data and concluded that BRT outperformed these in this instance [20].

#### Hyperparameter Tuning and Goodness of Fit Evaluation

The R package caret [32] was used to determine the hyper-parameters for the BRT model and split the dataset into training data to train the model and a test set to validate the predictive performance of the created model on unseen data. The goodness of fit of the model was evaluated using the root mean square error (RMSE). The results are summarised in Table 3. The final suggestions of the hyper-parameter tuning process were number of trees (n.trees = 2500), interaction depths between nodes in the tree branches (interaction.depth = 5), learning rate or shrinkage (shrinkage = 0.01) and a set number of minimum observations in the splitting node (n.minobsinnode = 10).

The BRT model was fit using the gbm package [33]. We set the formula parameter to “slope coefficents ∼ latitude + longitude” to describe the model, set the hyperparameters as described above and held all other parameters at their default values. The computational environment was the R statistical modelling software version 3.3.3 [25] running on a DELL laptop inside Windows 7 SP1 (64-bit Operating System, x64-based processor) on a 2.60 GHz Intel i7 CPU with 16 GB of RAM. All of the plots were generated in the R programming language [25].

## 4. Results

### 4.1. BRT Predictions

#### 4.1.1. Overall Results of the Entire Data Set

**Figure 7:**
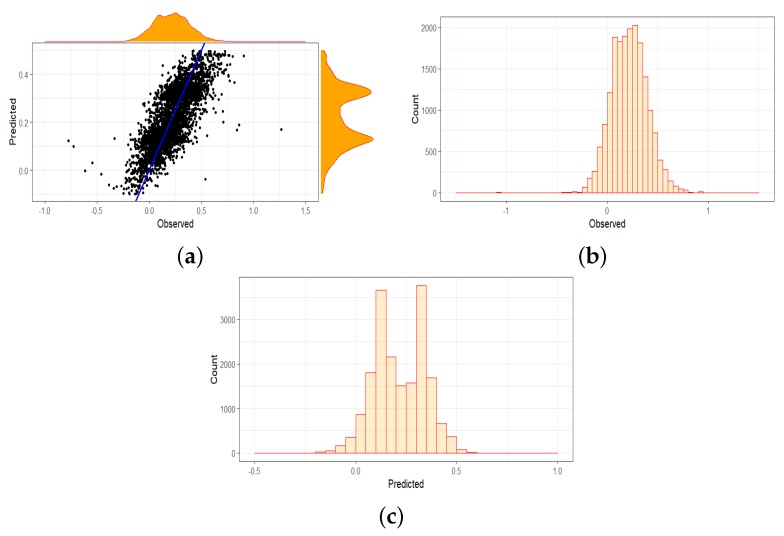
Data set showing 30 years in (**a**) marginal effects; (**b**) histogram of observed values and (**c**) of predicted values. (**a**) the BRT performance on predicting the slope coefficients strongly relate with the observed values; (**b**) histogram showing distribution of observed values; and (**c**) histogram showing distribution of predicted values.

The marginal plot in Figure 7a shows a strong linear relationship between the observed values and the corresponding predicted values obtained under the BRT model. It also shows the marginal distributions of the two sets of values, reflecting a relatively normal distribution for the observed values and a bimodal distribution for the predicted values. This is also shown in the histograms in Figure 7b,c. We can see somewhat unexpected slightly positive trends in the quantity of green vegetation cover in our study area throughout the years. This phenomenon is not unusual for tree-based models.

#### 4.1.2. Decadal Analyses

**Figure 8:**
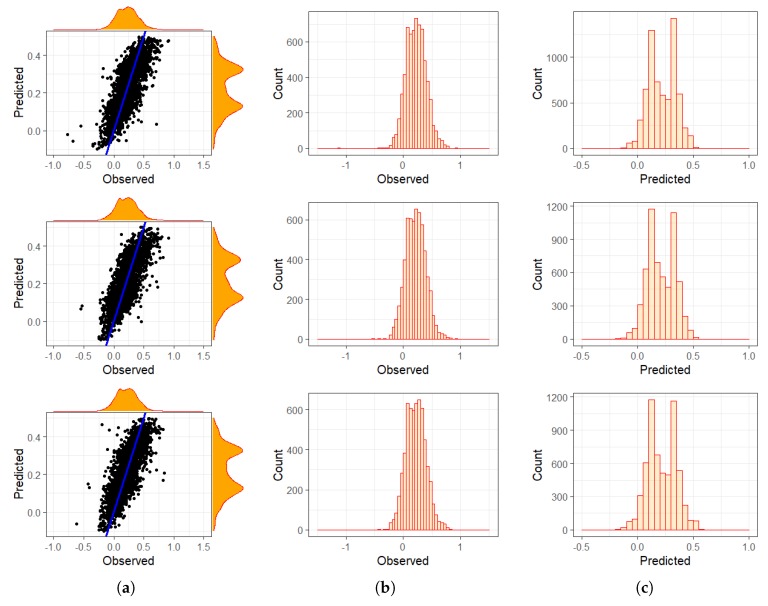
Plots of the decades showing (**a**) marginal effects; (**b**) histogram of observed values and (**c**) of predicted values. The top panel shows the decade starting at 1987, the middle panel starting at 1997, and the last panel starts with 2007.

The linear model captures only an overall monotonic rate of change over all 30 years and can not detect changing trends over time. To overcome this, we subdivided the 30 years into three 10-year time frames to investigate if changing trends within the decades could be detected individually. Figure 8 shows the comparison of our results and demonstrate that there is no significant difference. The analysis over three 10-year time frames showed a very similar temporal trend and a slightly increasing vegetation cover as in using the whole data set covering 30 years demonstrated in Figure 7. The scatterplot shows a strong positive correlation between the predicted values and the original measurements. You can see that BRT has the tendency to under- and over-predict extreme values demonstrated here on the predicted values following along the blue line of equality.

The decade-specific models displayed a similar fit to the overall model, with respect to RMSE (Table 3).

#### 4.1.3. Segmented Areas

In addition to splitting the data into decades, we used all 30 years and divided our study area into four even segments, namely upper left corner, lower left corner, upper right corner, and lower right corner. Altogether, the splits resulted in eight scenarios comprising (1) the whole data set described in Section 4.1.1, (2) three decades described in Section 4.1.2 and (3) four segmented areas of the whole data set described here. The overall model fit of all eight scenarios is shown in Table 3.

**Figure 9:**
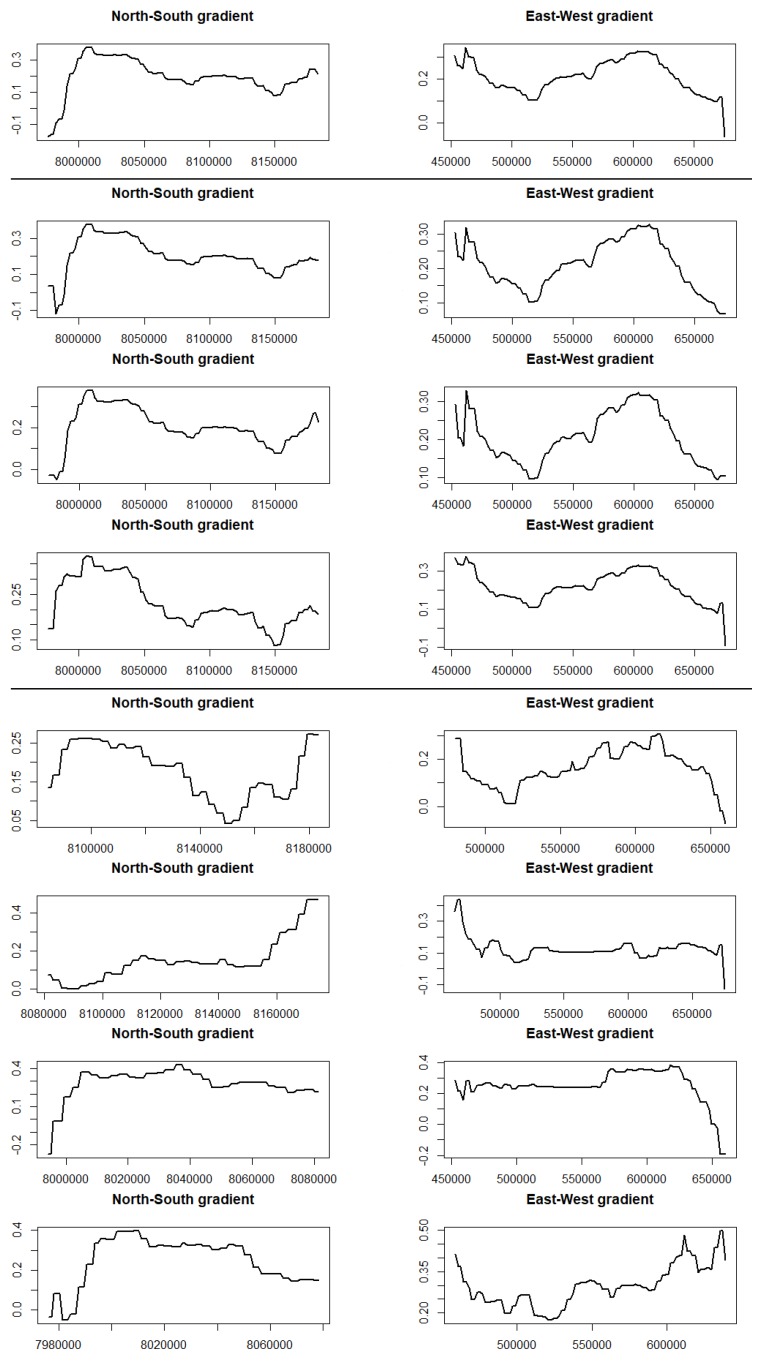
Partial dependency plots (PDP) of all eight scenarios in the order from top to bottom. All 30 years, first 10 years, middle 10 years, last 10 years, segment 1 (**upper left**), segment 2 (**upper right**), segment 3 (**lower left**), and segment 4 (**lower right**). On the left, there is the PDP of the latitude (North–South gradient) and the right shows the longitude (East–West gradient).

Partial dependency plots (PDP) are graphical visualizations of the marginal effect of our latitude and longitude covariates on the predicted response; here, the extracted slope coefficients from the linear model described in Section 3.1.1. Figure 9 shows all eight scenarios. On the left-hand side, we can see PDP showing the longitude. The general pattern shows an increase starting at the lower left corner at about the geographic coordinate of $8,000,000^{\circ }$ reaching its peak and then decreasing slowly again. The only exception can be seen on segment upper right corner. On the right-hand side, we see the latitude starting high at $450,000^{\circ }$ and falling and increasing again at $520,000^{\circ }$ with the exception of the lower left corner. Please see Figure 4 for further details on the categories of slope coefficients and their associated *p*-values with geographic coordinates.

To investigate the model fit, we used the Root Mean Square Error (RMSE) as shown in Table 3. All eight scenarios were created using 80% of data for training the model and the remaining 20% of data for testing the model performance on unseen data. The table shows the RMSE for the predicted values on the test data.

### 4.2. Relative Influence

The spatial pattern of the trend values over the case study image can be further assessed by evaluating the relative influence of each of the covariates, latitude and longitude, in the BRT analysis. The North–South gradient was slightly more influential than the East–West gradient. This pattern persisted in the decade analyses as well (Table 4).

## 5. Discussion

In this paper, we have proposed a two-step method for evaluating the spatial patterns of linear trends across a landscape, based on the geographic covariates latitude and longitude. The relative importance of these covariates, combined with the trend estimates themselves, can provide a deeper understanding of environmental impacts on the target response. For instance, in the case study considered here, the analyses allow insight into whether climate variability appears to have little to no impact on the existing green vegetation our study area. In Figure 2a, we show the distribution of green vegetation fractions in boxplots covering 30 years and visualising the inter quartile range, minimum and maximum values. We can see an increase in the median, especially in the years 2011 and 2014, the highest fraction of green vegetation of 70% and higher. This is surprising because, in many studies, a general trend of desertification in semi-arid regions around the world could be found. However, our results demonstrate that 84.48% of all extracted slope coefficients show a neutral to a slightly positive trend in green vegetation as shown in Table 2.

We conducted a temporal and spatio-temporal investigation on one overall data set or on three data sets covering one decade each to get a better understanding if there are seasonal patterns that will not be captured by the overall 30-year time frame. Our findings are demonstrated in Figure 7 and Figure 8. The RMSE errors listed in Table 3 indicate that there is no significant influence in dividing the data set to improve prediction accuracy. In addition, it can be seen that BRT under-predicts the slope coefficient when using geographic coordinates as spatial gradients.

By plotting the *p*-values using their geographic coordinates, we can demonstrate a spatial trend of significant strong *p*-values associated with the extracted slope coefficients as demonstrated in Figure 5. Furthermore, we demonstrate a stronger influence of the longitude coordinates in explaining our response variable as demonstrated in Table 4. To get insight into temporal and spatio-temporal trends, we split up the FCover scene and investigate several scenarios, namely the whole data set, the three decades and 30 years in four even segments of the FCover scene. We investigated if there are spatial trends in the slope coefficients and trends of green vegetation in each scenario. Figure 9 shows the influence of latitude and longitude in all eight scenarios as partial dependency plots. We can clearly see that each segment and each decade differ from each other and affirm our approach in using consecutive time intervals to investigate spatial green vegetation trends individually to get spatio-temporal insight into the amount of green vegetation fractions and how the greenness developed over space and time.

Using a linear model to extract slope coefficients allows formal, statistical investigation of the vegetation trends and associating *p*-values. However, it only considers trends as a linear monotonic trends of green vegetation. We tried to overcome this by dividing the data set into three decades, but it has been demonstrated that it did not improve prediction accuracy substantially. Furthermore, no turning points or extreme events were taken into consideration that would have described changes in green vegetation fractions since the linear approach could not have detected them. As seen in Figure 8, we used this to split the data into three decades, which shows that there were no other significant trends captured. We only used geographic surrogate gradients without adding any other environmental covariates to the BRT model. In addition, no further testing using other FCover scenes of a different Landsat footprint was taken to determine if the results are restricted to our location only.

There are many generalisations of the approach presented here. For example, while this paper has intentionally focused on a single analytic method for each of the two steps in the proposed approach, it is clear that these methods could be replaced by any one—or indeed a number—of a wide variety of statistical machine learning methods designed for estimation and/or prediction. For example, instead of the linear regression and BRT approaches illustrated here, one could consider other regression models that capture temporal and spatial correlation (e.g., exponentially weighted moving average models, Markov random field models, respectively) or other nonlinear models such as neural networks or support vector machines. It is also valuable to look at the literature in other domains that evaluate and compare these and other methods for spatial and temporal estimation and prediction, such as [34,35,36]. Generalising in another direction, although this paper has focused on a single output from the first step (the estimated regression coefficient) and used this as a univariate response for the analysis in the second step, a multivariate approach could be adopted whereby the outputs from the first step (and inputs for the second step) are the regression estimates and their associated standard errors and/or RMSE, or estimates of multiple coefficients in a multiple regression, or parameter estimates and associated goodness of fit estimates from an alternative supervised learning method such as a neural network.

## 6. Conclusions

In this study, we demonstrated that a localised and quantitative distribution of temporal and spatio-temporal trends of green vegetation cover can be predicted using BRT. All together, eight scenarios have been investigated, namely the whole data set covering 30 years, then three data sets covering a decade each, then the four quadrants of the image over all years. We showed that the prediction of location-based trends of green vegetation achieved good results by using the RMSE as goodness of model fit by combining a linear model and BRT. The extracted slope coefficient and *p*-values were categorised and further analysed by their direction of their increase of the quantity in green vegetation and their associated statistical significance through the *p*-values. A limitation can be found that 84% of the slope coefficients were positive but most were associated with non-significant *p*-values. In our paper [12], we concluded that a North–South gradient is dominating over the East–West gradient in predicting the quantity of green vegetation fractions used in a spatial context. Here, we are using the same data and we can see that the North–South gradient does not contribute to the rate of change in green vegetation and its influence for temporal trends based on the three decades. Our results confirm the results of the author [13], where they concluded that latitude and longitude can be used to explain the spatial variability in the distribution of C3 and C4 grass along North–South and East–West gradients. In analysing 30 years as a spatio-temporal aspect in the four segments demonstrated in Table 4, we show a decrease of the influence of the North–South gradient and an increase of the East–West gradient as the relative influence of predicting vegetation trends. By analysing the data using either the whole data set of 30 years, three decades or four segments that show 30 years in each quadrant, we can conclude that, in the shorter time frames, no temporal trends were observed and the overall linear trend of 30 years seems sufficient.

## Figures and Tables

**Table 1:** Descriptive statistics of the green vegetation fractions for the whole data set covering 30 years.

Min.	1st Qu.	Median	Mean	3rd Qu.	Max.
0.00	11.64	17.03	18.37	23.16	73.92

**Table 2:** Six categories of slope coefficients with corresponding numbers of observations in the data set, and their overall representation/contribution as percentages in the case study.

Slope Coefficient Categories	Observations	Percentages %
slope coefficient > 1	14	0.02%
slope coefficient >= 0.5 and slope coefficient < 1	5088	5.44%
slope coefficient >= 0 and slope coefficient < 0.5	79032	84.48%
slope coefficient >= −0.5 and slope coefficient < 0	9364	10.01%
slope coefficient >= −0.5 and slope coefficient < −1	30	0.03%
slope coefficient < −1	19	0.02%

**Table 3:** Root Mean Square Error (RMSE) on the test data using all 30 years, first 10 years, middle 10 years and last 10 years and in four segmented areas comprising a 30-year time frame.

Scenario	RMSE
All 30 years	0.1150
First 10 years	0.1112
Middle 10 years	0.1214
Last 10 years	0.1063
Four segments
1—Upper left	0.1076%
2—Upper right	0.0915%
3—Lower left	0.1112%
4—Lower right	0.1265%

**Table 4:** Relative influence of the longitude in explaining the response using all 30 years, first 10 years, middle 10 years, last 10 years, and in all four segments of the FCover scene.

Scenario	North–South Gradient
All 30 years	56.77%
First 10 years	57.04%
Middle 10 years	55.68%
Last 10 years	57.67%
Four segments	
1—Upper left	34.63%
2—Upper right	47.71%
3—Lower left	40.79%
4—Lower right	43.24%
